# Characterization of Mechanical Properties and Grain Size of Stainless Steel 316L via Metal Powder Injection Molding

**DOI:** 10.3390/ma16062144

**Published:** 2023-03-07

**Authors:** In-Seok Hwang, Tae-Yeong So, Do-Hoon Lee, Chang-Seop Shin

**Affiliations:** 1 Department of Biosystems Engineering, Chungbuk National University, Cheongju 28644, Republic of Korea; 2 Industrial Materials Processing R&D Department, Korea Institute of Industrial Technology (KITECH), Incheon 21999, Republic of Korea

**Keywords:** metal powder injection molding, binder jet printing, stainless steel 316L, mechanical properties, microstructure

## Abstract

The metal powder injection molding process is completed by mixing a metal powder and a binder, performing an injection molding and degreasing process, and then performing a sintering process for high density. The disadvantage of metal powder injection molding is that defects occurring during the process affect mechanical properties, which are worse in mechanical properties than in products manufactured by cold-rolling. In this study, the mechanical properties and microstructure of stainless steel 316L manufactured by the metal powder injection molding process were analyzed. Mechanical properties such as density, tensile strength, and fatigue life were analyzed. The density was measured using Archimedes’ principle, and a relative density of 94.62% was achieved compared to the theoretical density. The tensile strength was approximately 539.42 MPa and the elongation to fracture was approximately 92%. The fatigue test was performed at 80% of maximum tensile strength and a stress ratio of R = 0.1. The fatigue life was found in 55% (297 MPa) of maximum tensile strength that achieved 10^6^ cycles. The microstructure was observed through scanning electron microscope after etching, and as a result, the average grain size was 88.51 μm. Using electron backscatter diffraction, inverse pole figure map, image quality map, and kernel average misorientation map of the specimen were observed in three different areas which were undeformed, uniformly deformed, and deformed. Based on these results, it is expected that research is needed to apply the metal powder injection molding process to the manufacture of agricultural machinery parts with complex shapes.

## 1. Introduction

Metal powder injection molding (MIM) is a modification of the plastic injection molding (PIM) process. In MIM, a significant volume fraction of plastic in PIM is replaced by a fine metal powder [1]. MIM has penetrated manufacture applications in diverse industries, such as medical, automobile, and aerospace area, due to the manufacturability of the net shape without machining [2]. The sintering procedure in MIM processes makes final products have full density. Thus, MIM products have high strength, precision, and complexxity compared with products produced by other processes such as die casting, investment, sand casting, and forming routes [3]. As of 2011, MIM is growing at 14% per year and the MIM products were globally valued at approximately 1 billion USD [3]. MIM can mass-produce parts of complex shapes, thus, many studies have been conducted globally. However, in South Korea, the study of MIM has not yet been addressed [4].

Stainless steel (SS) 316L is one of the materials with excellent corrosion resistance, weldability, and mechanical properties [5,6,7]. The mechanical properties and microstructures of SS 316L depend on the manufacturing processes. Still, research on the mechanical properties and microstructures needs to be performed according to manufacturing processes so that MIM processes can be utilized in more diverse metal industry products, such as agricultural machinery including agricultural tractors, harvesters, and transplanters.

Electron backscatter diffraction (EBSD) allows us to construct several important maps representing characteristics such as inverse pole figure (IPF), image quality (IQ), kernel average misorientation (KAM), etc. [8]. These maps are helpful to determine the grain orientation for the different phases, quantify the local strain distribution, and determine strain or deformation in the crystal lattice structure [9]. The IPF map is colored according to the crystal plane normal direction (ND) aligned with a specified specimen direction. The color scheme used has red for (001), green for (101), and blue for (111) [10]. The IQ map is one of the common maps that consist of EBSD data. Additionally, the IQ map is the metric system describing the quality of a diffraction pattern [11]. The KAM map is one of the misorientation maps. In this map, the arithmetic mean of the scalar misorientation between groups of pixels, or kernels, is calculated and mapped [12]. The KAM map shows areas with a high density of defects and, as a mean value for the entire scan, may represent material strain history [13].

In this study, the mechanical properties were compared between cold-rolled and MIM SS 316L and then the microstructure was analyzed via scanning electron microscope (SEM). Additionally, the grain size was obtained through the American Standard Test and Method (ASTM) [14] along with measurements using the SEM device. Additionally, the Hall–Petch relation [15] is briefly discussed through grain size and mechanical properties.

## 2. Materials and Methods

**Figure 1:**
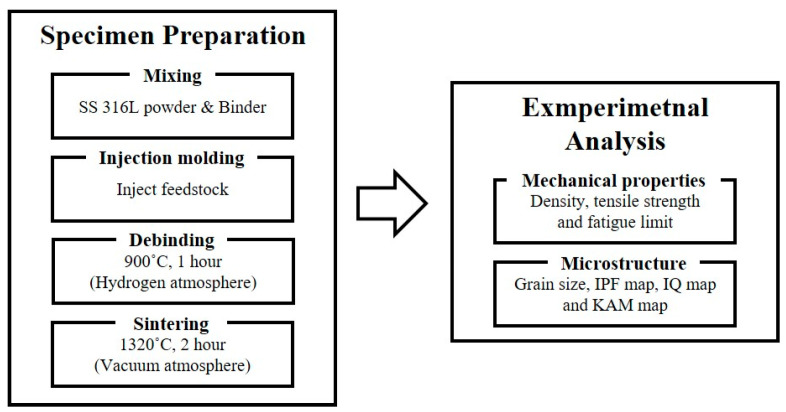
The outline of characterization of SS 316L.

**Figure 2:**
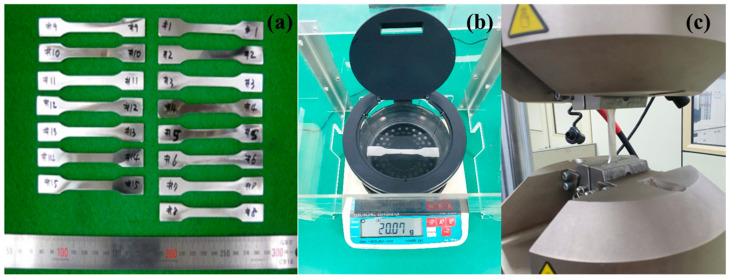
The process of measuring mechanical properties: (**a**) specimens produced by MIM process; (**b**) electronic densimeter (md-200 s); (**c**) servohydraulic fatigue testing system (Instron 8801).

The overall outline of this research is shown in Figure 1. The specimen was produced via MIM process using the feedstock of SS 316L and binder blended through roll mixing. We produced the specimen according to ASTM standard [16]. To prevent defects such as crevices, the debinding process, which removes the binder from the injection-molded articles mixed from the injection molder, is an inevitable process. The debinding process was kept at 900 °C for an hour in a hydrogen atmosphere. As a final process of MIM, the sintering is the heat treatment process to ensure that the final product obtains the designed mechanical properties. The sintering process was kept at 1320 °C for two hours in a vacuum atmosphere. In this study, we produced fifteen SS 316L specimens from MIM as shown in Figure 2a. Additionally, the chemical composition of SS 316L, water-atomized powder, is shown in Table 1.

The densities of specimens were measured using electronic densimeter (Grass Valley, CA, USA) using Archimedes’ principle as shown in Figure 2b. Additionally, the servohydraulic fatigue testing system (INSTRON 8801) was used as shown in Figure 2c.

The tensile test was performed to the fracture at the constant speed of 5 mm/min for three specimens each. The fatigue test, the stress ratio of which is 0.1, was performed at completely reversed cyclic stresses. The maximum stresses of the fatigue tests were set based on the ultimate tensile strength obtained in the tensile test. These stresses were set at 80% (432 MPa), 75% (405 MPa), 72.5% (391 MPa), 70% (378 MPa), 67.5% (364 MPa), 65% (351 MPa), 62.5% (337 MPa), 60% (324 MPa), 57.5% (310 MPa), and 55% (297 MPa) of the ultimate tensile strength. The fatigue limit was determined as the stress value applied when the number of loading cycles goes beyond 10^6^ in this study.

**Figure 3:**
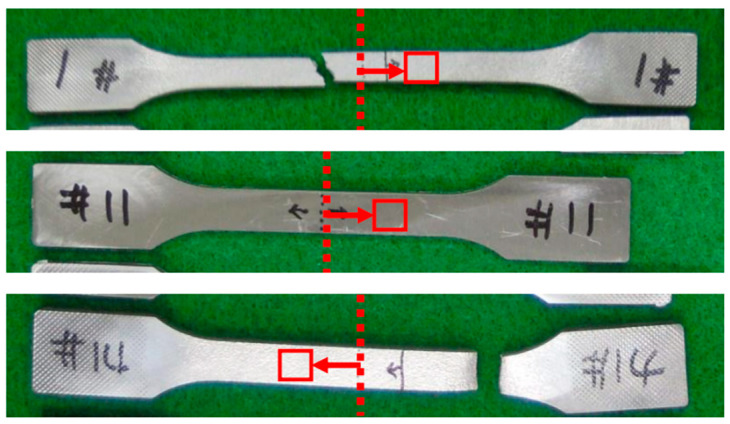
Specimens of microstructure observation.

Three specimens were etched using the etchant composed of reagents shown in Table 2 and then the microstructures of those specimens were investigated at the point located 5 mm opposite to the fracture from the center of the specimen by scanning electron microscope (SEM, ZEISS, Jena, Germany) as shown in Figure 3. The specimens of number 1 and 14 are the ones after the tensile and fatigue test, respectively. The specimen of number 11 is the one before the test.

The electron backscatter diffraction (EBSD) analysis was performed using a field emission scanning electron microscope (FE-SEM, Hitachi SU500) with an EDAX EBSD (Velocity Super) system. The specimen surfaces were prepared by polishing until a final polish step with 0.02 μm colloidal silica suspension was used. The scanning step size is 1 μm with an accelerating voltage of 20 kV. The raw EBSD data were analyzed using the TSL OIM software. EBSD data with a confidence index (CI) less than 0.1 were excluded.

Grain size was analyzed by planar grain size based on the intercept method delineated in ASTM standard [14], which means two-dimensional grain sections revealed by the sectioning plane. At first, the grain size number was determined by summing the intercepted lines between the grain boundaries and the drawn lines. The intercept method is faster than the planimetric method for the same level of precision when obtaining grain size, because an accurate count can be obtained without the need for marking off intercepts or intersections [14].

(1) $$\bar{l}=\frac{L_{T}}{PM}$$

In Equation (1), P is the number of grain boundary intersections with a test line, and $L_{T}$ is total length of test lines. $\bar{l}$ is the mean lineal intercept length, and M is magnification used. G is ASTM grain size number, which is determined using Equation (2) and converted to the grain size.

(2) $$G=\left(-6.643856\log_{10}\bar{l}\right)-3.288$$

## 3. Results and Discussion

### 3.1. Density

**Figure 4:**
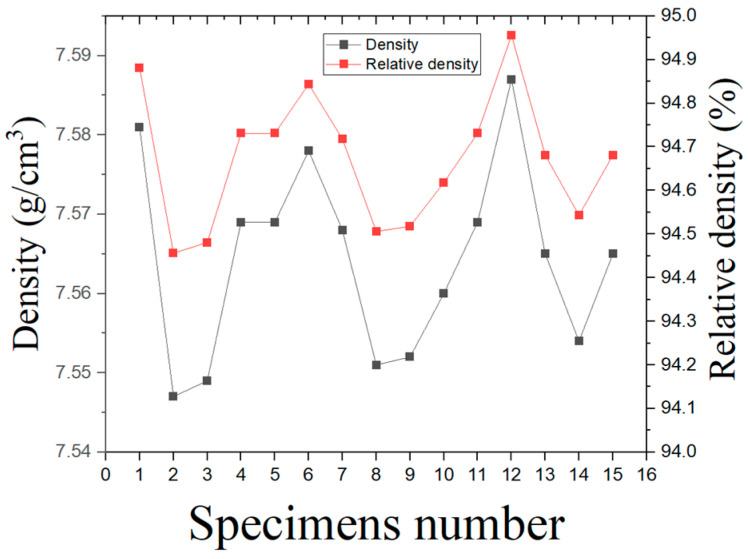
The density and relative density of the SS 316L specimens produced by metal powder injection molding.

The maximum density in the MIM process is obtained after the sintering process. The average density of specimens measured using Archimedes’ principle was 7.56 $g/{\mathrm{cm}}^{3}$. The relative density compared to the theoretical density, 7.99 $g/{\mathrm{cm}}^{3}$ [2], was 94.62% on average as shown in Figure 4. Thus, the specimens produced in this study seem to have more than 5% porosity.

It is suggested that the relative density differences observed among the specimens result from the development of porosity, oxide particles, and other factors within the specimens [17]. In addition, the porosity formation varies due to the melting and cooling behavior of powder particles, resulting in a higher number of porosities in low-density specimens than in high-density specimens [18]. The disadvantage of the MIM process is that it has a lower density than conventional processes. The density of the MIM process is greatly affected by the conditions of the binder composition, and the debinding and sintering processes [19,20,21]. The shape and size of the particles in a powder can influence its packing density and flowability. Insufficient packing density or non-uniform distribution of particle sizes can lead to a decrease in relative density. In the MIM process, a binder is mixed with metal powders to create a feedstock that can be shaped into the desired form. During binder removal, the binder is heated and vaporized, which results in the formation of a porous structure. The incomplete removal of the binder can lower the relative density due to voids or defects. Furthermore, during sintering after binder removal, the metal particles shrink and densify. However, the existence of defects or voids can disturb full densification, resulting in a reduction in the relative density. Thus, a lot of research is being conducted for full density [22,23,24]. The porosity may be reduced by the post-treatment process [25].

### 3.2. Stress–Strain Diagram

**Figure 5:**
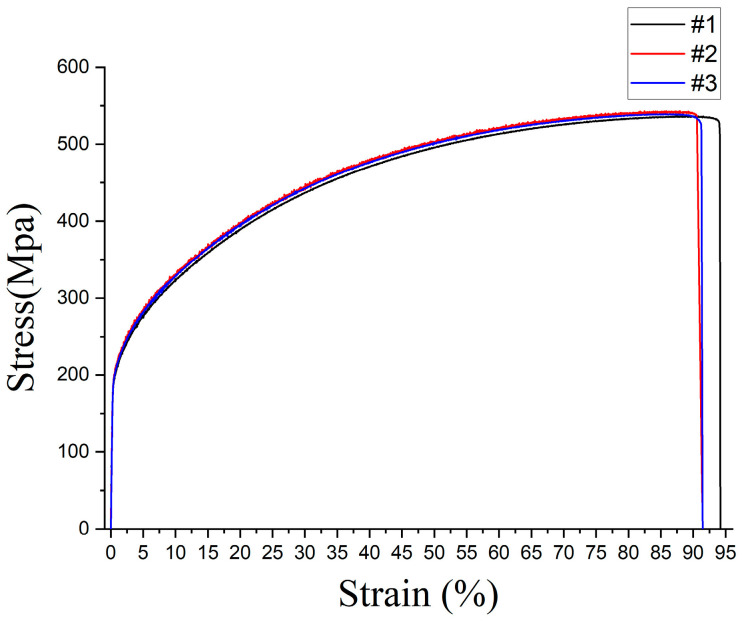
Stress–strain curve of SS 316L produced by metal powder injection molding.

The average value of tensile strength obtained from the tensile tests is 539 MPa; 536 MPa for specimen 1, 543 MPa for specimen 2, and 539 MPa for specimen 3 as shown in Figure 5. The average tensile strength of the MIM SS 316L specimen was lower than the tensile strengths of the cold-rolled specimen (620–795 MPa) [26] and the hot-rolled specimen (580 MPa) [27], which were 13% to 32% lower than the cold-rolled specimen and 7% lower than the hot-rolled specimen. Two mechanisms were identified as the primary causes of reduced mechanical properties in MIM SS 316L specimens due to the presence of pores: (1) reduction in the area where stress is applied, and (2) polygonal pores causing a notch effect, which could potentially lead to early material failure [28]. The elongation to the fracture of the MIM specimen was measured to be 92%. which was increased compared to that of the cold-rolled specimen (30%) [26] and the hot-rolled specimen (89%) [27], which was 206% higher than the cold-rolled specimen and 3% higher than the hot-rolled specimen. Additionally, as the sintering temperature increases, the porosity decreases, leading to an increase in ductility. However, sintering at excessively high temperatures can result in a decrease in ductility due to abnormal grain growth and the precipitation of carbides in the grain boundary [28]. The results indicate that the sintering temperature of 1320 °C was the proper point to achieve high ductility of 92% in the MIM SS 316L specimen. Thus, it was confirmed that the inverse proportionate relationship between the tensile strength and the elongation to the fracture was also applicable to the MIM SS 316L specimen in this study. Additionally, the fracture point of the specimen became the ultimate tensile strength in this study.

### 3.3. Fatigue Life

**Figure 6:**
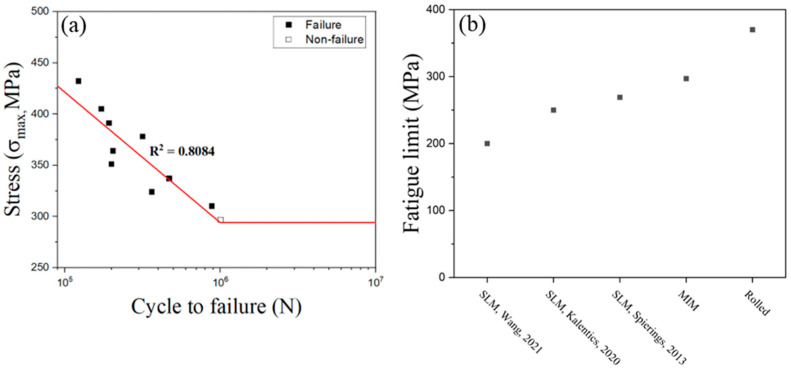
(**a**) Stress–life curve of SS 316L produced by metal powder injection molding. (**b**) Fatigue limit measurement results reported in the literature and in this work for SS 316L [29,30,31].

The fatigue testing (stress ratio was 0.1) was performed for 10 specimens to determine the endurance limit, which is also called the fatigue limit. The loading stress was started from 80% of tensile strength, 432 MPa, and reached up to 55%, 297 MPa, which can be the endurance limit as shown in Figure 6a. The endurance limit of MIM SS 316L followed the general range between 0.4 and 0.6 of tensile strength. The fatigue limit of the selective laser melting (SLM)-fabricated SS316L was reported to be lower than that of the rolled material [29,30,31]. The fatigue limit of the MIM SS 316L was higher than that of the SLM-fabricated SS 316L and lower than that of rolled SS 316L as shown in Figure 6b [29,30,31]. The difference in fatigue limit between MIM SS 316L, SLM-fabricated SS 316L, and rolled SS 316L can be explained by the microstructural characteristics of each material. In the case of SLM-fabricated SS 316L, the microstructure is known to exhibit a columnar grain structure with high anisotropy, which can lead to stress concentration and the initiation of microcracks during fatigue testing [32]. However, the rolling process of SS 316L results in a decrease in pores and the formation of a uniform and fine microstructure. This microstructure contributes to increased resistance against fatigue crack initiation and propagation, which results in a higher fatigue limit compared to SLM-fabricated and MIM SS 316L. In the case of MIM SS 316L, sintering at an appropriate temperature of 1320 °C results in a uniform microstructure with minimal porosity, which leads to higher ductility and fatigue limits than SS 316L fabricated by SLM.

### 3.4. Microstructure

**Figure 7:**
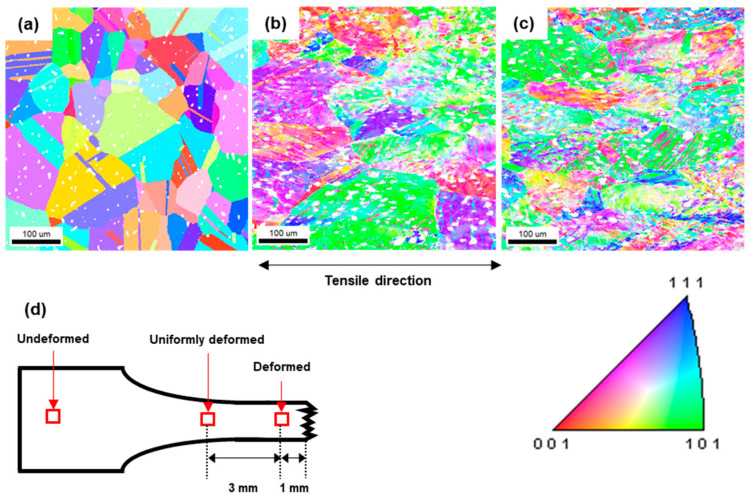
EBSD results in inverse pole figure map along the deformation gradient; (**a**) undeformed; (**b**) uniformly deformed; (**c**) deformed; (**d**) schematic of the observed area.

The deformation of the microstructures of the specimen by tensile deformation is analyzed using EBSD. The specimen was observed in three different areas, which were undeformed, uniformly deformed, and deformed as shown in Figure 7d. The inverse pole figure (IPF) maps of each area are presented in Figure 7a through Figure 7c. The area with a confidence index (CI) of less than 0.1 appears white, indicating the presence of pores and cracks. With increasing deformation, the degree of color gradation within the grains increases. It indicates that the grain orientations are uniform in the initial stage; however, as the deformation increases, misorientation within the grains increases.

**Figure 8:**
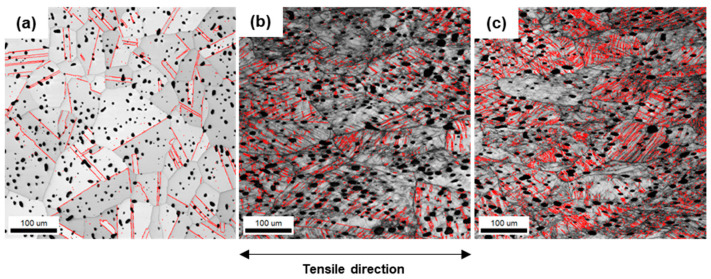
Image quality maps (red: twin boundaries) along the deformation gradient; (**a**) undeformed; (**b**) uniformly deformed; (**c**) deformed.

Figure 8 shows the image quality (IQ) maps that exhibit the quality of the EBSD patterns in grayscale. The black spots indicate the micropore of the specimens. The perfect crystal area shows a high IQ (light) value, whereas the defect areas are low (dark) [33]. It is known that a deformed microstructure produces a lower IQ value [34]. The results of IQ maps show that the undeformed grains become difficult to distinguish after the deformation because the IQ value is lower with increasing deformation. The low IQ values in the deformed microstructure indicate that severe plastic deformation has been applied to the specimen. As the deformation increases, the deformation bands increase, and the austenite grains become fragmented. Figure 8a shows the twin boundaries within the austenite grain, and the density of the twin boundaries increases with increasing deformation as shown in Figure 8b,c. It has been reported that deformation twins form during plastic deformation with austenitic SS having low stacking fault energy (SFE), thus the twin density increases with increasing deformation [35]. The deformation bands and twins are formed during plastic deformation to fragment the austenite grains and suppress the dislocation movement. Thus, it can have a positive effect on enhancing the mechanical properties.

**Figure 9:**
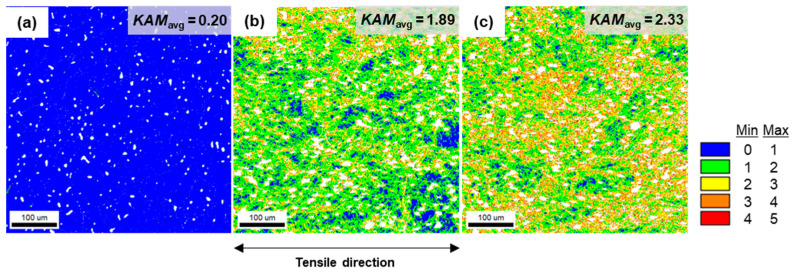
Kernel average misorientation (KAM) maps along the deformation gradient, (**a**) undeformed, (**b**) uniformly deformed, and (**c**) deformed.

Figure 9 show the color-coded kernel average misorientation (KAM) maps to quantify the local strain distribution in specimens with different deformation gradients. The white spot indicates the micropore of the specimens. The blue color indicates a value less than 1°, while the green indicates a value range from 1° to 2°, the yellow from 2° to 3°, the orange from 3° to 4°, and the red from 4° to 5°. The high KAM values indicate larger plastic deformation or accumulation of the dislocations along high-angle grain boundaries [36].

Figure 9a–c show the average KAM values of the specimen with the deformation gradient, and it is indicated that the average KAM values increased as the deformation gradient increased. As shown in Figure 9a, the average KAM value of the undeformed area was very low, i.e., 0.2, showing that the area was practically strain-free. However, Figure 9b shows that dislocation was accumulated in the uniformly deformed area, resulting in a significant increase in the average KAM value from 0.2 to 1.89. This result exhibits that a large density of dislocations was formed at a relatively early stage of deformation. Furthermore, the high dislocation density acts as a stable and soft barrier, suppressing the dislocation migration for strength while guaranteeing a continuous plastic flow which preserves ductility by transmitting the partial dislocation.

**Figure 10:**
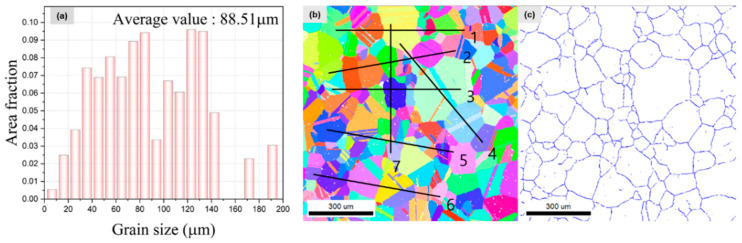
Grain size: (**a**) grain size distribution; (**b**) intercept by lines through grain boundaries; (**c**) grain boundaries.

Grain size distribution of the specimen before deformation is shown in Figure 10a. The twin boundaries were ignored when evaluating the ASTM grain size number because twin boundaries belong to the grain according to the ASTM standard [14]. The grain size was almost same as the one evaluated by the intercept method (G = 3.86, average diameter = 94.27 μm) by lines through the grain boundaries and the calculated one by the EBSD (G = 4.04, average diameter = 88.51 μm). Based on the nearly identical results obtained through the two methods, the EBSD measurement for grain size was replaced with the intercept method using SEM images to measure the grain size of specimens including tensile fracture, as-received, and fatigue fracture.

**Figure 11:**
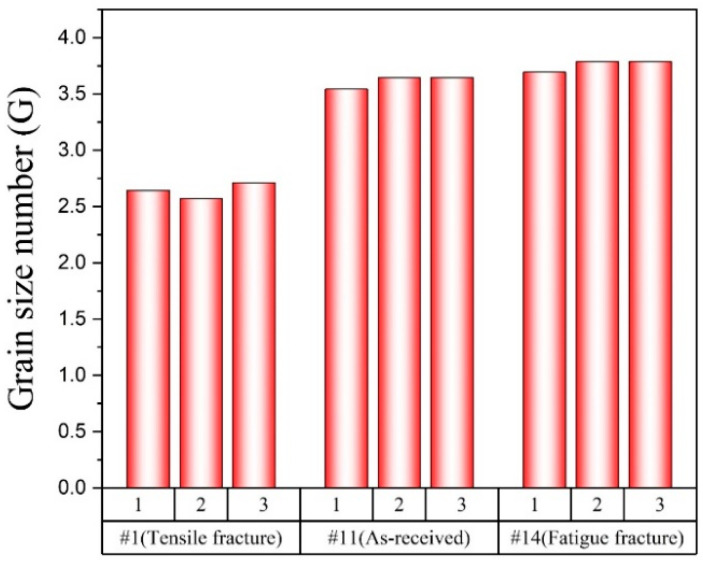
Grain size number of specimens.

Figure 11 shows the grain size number of each of the three SEM images for each specimen (#1, #11, #14) using the intercept method. The ASTM grain size number of the tensile fracture specimen was lower than the as-received MIM SS 316L specimen. Tensile stress on the specimen reduces its cross-sectional area while increasing axial stress and strain. As a result, the grains within the material deform and restructure, leading to the generation of new grains and a decrease in mean grain size [37]. However, the ASTM grain size number of the fatigue fracture specimen was higher than the one of the as-received MIM SS 316L specimen as shown in Figure 11. During fatigue testing, materials are subjected to cyclic loading, which can lead to the formation of microcracks within the material. The tip of a microcrack generates the migration of grain boundaries due to stress concentration [38]. As a result of grain boundary migration, the coalescence of grains occurs, resulting in an increase in the average grain size of the material.

**Figure 12:**
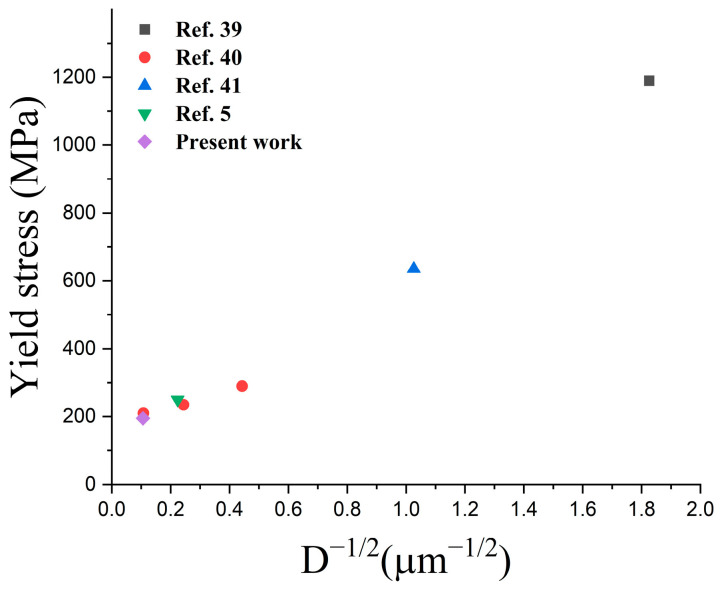
The effect of grain size versus the yield strength based on Hall–Petch relationship of SS 316L.

Figure 12 shows the effect of grain size on the yield strength based on the results in the literature [5,39,40,41] and of this study. As the grain size decreased, the yield strength increased. Thus, we confirmed that even with a 95% relative density and a microstructure with porosity, the particle size and yield strength follow the Hall–Petch relationship [15]. The results demonstrate that the appropriate grain size for the sintering temperature of 1320 °C and the increase in twin boundaries and dislocation density in the microstructure during deformation are the strengthening factors.

## 4. Conclusions

We compared the mechanical properties between cold-rolled and MIM SS 316L and analyzed microstructure along with grain size using the intercept method and EBSD calculations. The following conclusions were obtained:

1. The average density of MIM SS 316L specimens is 7.56 g/cm^3^. The relative density measured was 94.68% of the theoretical density.
2. The average tensile strength (539 MPa) of the MIM SS 316L specimen was lower than the tensile strength of the cold-rolled (620–795 MPa) and hot-rolled (580 MPa) specimens. The elongation was 92%, which was higher than the elongation (30%, 89%) of the specimen manufactured by cold rolling and hot rolling. Because of its high elongation, it has excellent impact resistance. The fatigue limit was measured at 297 MPa (55% of tensile strength), which is higher than SLM SS 316L and lower than cold-rolled SS 316L.
3. With increasing deformation, the austenite grains become fragmented due to increases in misorientation, the density of the twin boundary, and the deformation band. Accordingly, these behaviors suppressed the dislocation movement during tensile stress, which thereby enhanced the mechanical properties.
4. The average KAM value was a significantly increased at the relatively early stage of deformation. This result shows that a high density of dislocations was formed, which act as a soft barrier that restricts dislocation migration for increased strength, while also allowing for a continuous plastic flow to maintain ductility by transmitting partial dislocations.
5. The grain size was almost the same between the one evaluated by the intercept method by lines through the grain boundaries and the one calculated by the EBSD. A precision of better than 0.25 grain size units was obtained.

## Figures and Tables

**Table 1:** Chemical composition from the powder manufacturer.

Element	C	Si	Mn	P	S	Ni	Cr	Mo	Cu	Fe
**Mass %**	0.024	0.79	0.79	0.009	0.006	12.58	16.43	2.11	0.05	balance

**Table 2:** Composition of the etchant used to obtain the microstructure in this study.

Etchant		Time
Glycerol	45 mL	1 m 30 s ~2 m
Hydrochloric acid	15 mL	
Nitric acid	30 mL	

## Data Availability

Not applicable.
